# Variation in Case Exposure During Internal Medicine Residency

**DOI:** 10.1001/jamanetworkopen.2024.50768

**Published:** 2024-12-18

**Authors:** Andrew C. L. Lam, Brandon Tang, Chang Liu, Marwa F. Ismail, Surain B. Roberts, Matthew Wankiewicz, Anushka Lalwani, Daniel Schumacher, Benjamin Kinnear, Amol A. Verma, Fahad Razak, Brian M. Wong, Shiphra Ginsburg

**Affiliations:** 1 Department of Medicine, University of Toronto, Toronto, Ontario, Canada; 2 Division of General Internal Medicine, Department of Medicine, University of Toronto, Toronto, Ontario, Canada; 3 Li Ka Shing Knowledge Institute, Unity Health, Toronto, Ontario, Canada; 4 Institute of Health Policy, Management and Evaluation, University of Toronto, Toronto, Ontario, Canada; 5 Schulich School of Medicine & Dentistry, Western University, London, Ontario, Canada; 6 Department of Pediatrics, Cincinnati Children’s Hospital Medical Center, Cincinnati, Ohio; 7 Division of General Internal Medicine, Department of Medicine, Unity Health, Toronto, Ontario, Canada; 8 Department of Laboratory Medicine and Pathobiology, University of Toronto, Toronto, Ontario, Canada; 9 Centre for Quality Improvement and Patient Safety, Temerty Faculty of Medicine, University of Toronto, Toronto, Ontario, Canada; 10 Division of General Internal Medicine, Sunnybrook Health Sciences Centre, Toronto, Ontario, Canada; 11 Division of Respirology, Department of Medicine, Mount Sinai Hospital Department of Medicine, Toronto, Ontario, Canada

## Abstract

**Importance:**

Variation in residency case exposure affects resident learning and readiness for future practice. Accurate reporting of case exposure for internal medicine (IM) residents is challenging because feasible and reliable methods for linking patient care to residents are lacking.

**Objective:**

To develop an integrated education-clinical database to characterize and measure case exposure variability among IM residents.

**Design, Setting, and Participants:**

In this cohort study, an integrated educational-clinical database was developed by linking patients admitted during overnight IM in-hospital call shifts at 5 teaching hospitals to senior on-call residents. The senior resident, who directly cares for all overnight IM admissions, was linked to their patients by the admission date, time, and hospital. The database included IM residents enrolled between July 1, 2010, and December 31, 2019, in 1 Canadian IM residency. Analysis occurred between August 1, 2023, and June 30, 2024.

**Main Outcomes and Measures:**

Case exposure was defined by patient demographic characteristics, discharge diagnoses, volumes, acuity (eg, critical care transfer), medical complexity (eg, Charlson Comorbidity Index), and social determinants of health (eg, from long-term care). Residents were grouped into quartiles for each exposure measure, and the top and bottom quartiles were compared using standardized mean difference (SMD). Variation between hospitals was evaluated by calculating the SMD between the hospitals with the highest and lowest proportions for each measure. Variation over time was assessed using linear and logistic regression.

**Results:**

The integrated educational-clinical database included 143 632 admissions (median [IQR] age, 71 [55-83] years; 71 340 [49.7%] female) linked to 793 residents (median [IQR] admissions per shift, 8 [6-12]). At the resident level, there was substantial variation in case exposure for demographic characteristics, diagnoses, volumes, acuity, complexity, and social determinants. For example, residents in the highest quartile had nearly 4 times more admissions requiring critical care transfer compared with the lowest quartile (3071 of 30 228 [10.2%] vs 684 of 25 578 [2.7%]; SMD, 0.31). Hospital-level variation was also significant, particularly in patient volumes (busier hospital vs less busy hospital: median [IQR] admissions per shift, 10 [8-12] vs 7 [5-9]; SMD, 0.96). Over time, residents saw more median (IQR) admissions per shift (2010 vs 2019: 7.6 [6.6-8.4] vs 9.0 [7.6-10.0]; *P* = .04) and more complex patients (2010 vs 2019: Charlson Comorbidity Index ≥2, 3851 of 13 762 [28.0%] vs 2862 of 8188 [35.0%]; *P* = .03), while working similar shifts per year (median [IQR], 11 [8-14]).

**Conclusions:**

In this cohort study of IM residents in a Canadian residency program, significant variation in case exposure was found between residents, across sites, and over time.

## Key Points

**Question:**

How does case exposure vary between residents within a large residency program, and how does case exposure vary across hospitals and over time?

**Findings:**

This cohort study included 793 internal medicine residents from 5 hospitals with 143 632 admissions over 10 academic years. Even in the same program, case exposure varied substantially between residents, hospitals, and over time across all 6 studied domains (patient volume, demographic characteristics, breadth of presentation, acuity, complexity, and social determinants of health).

**Meaning:**

The findings of this study suggest that training programs need methods of tracking case exposure and identifying interresident variation to train physicians to deliver high-quality care.

## Teaser

This cohort study evaluates case exposure variability among internal medicine residents in Canada using an integrated database that links trainees to the patients they treat.

## Introduction

Residency is a time of significant imprinting. Practices developed during residency persist long into independent practice.^1,2^ In surgical residencies, variations in patient volumes and breadth are linked to differences in technical competency, procedural length, and postoperative complications.^3,4,5^ In medical residencies, variation in case exposure is linked to differences in in-training examination scores, clinical decision-making concordance with supervising physicians, and postadmission mortality after transitioning to independent practice.^6,7,8,9^ Undoubtedly, variation in exposure during residency affects educational and clinical outcomes as well as readiness for future practice.^10^

Despite the importance of case exposure and its relationship to key outcomes, most residency programs lack systems to accurately track these metrics.^11,12^ Furthermore, systems to quantify and explore sources of variation among residents remain unavailable.^10^ Prior efforts to measure case exposure have typically relied on resident-reported case logs. However, these suffer from recall bias, missing data, and have coding error rates ranging from 19% to 47%.^13,14,15^ Furthermore, this adds cognitive and time burdens to residents, which may worsen the high rates of resident burnout.^16^

Automated systems tracking clinical decisions and outcomes for audit-and-feedback have been implemented for attending physicians and can improve care processes and patient outcomes.^17^ These systems have been used to develop quality improvement initiatives for conditions ranging from pediatric diabetic ketoacidosis to β-lactam use in surgery, with initiatives shown to influence physician practices.^18^ However, such systems for residents remain preliminary. An early example from Drake et al^19^ tracked the clinical encounters of 51 internal medicine (IM) residents throughout their training. However, this study only captured 1 dimension of case exposure (discharge diagnoses), derived data from 1 hospital, and had a limited number of residents.

In this study, we developed an integrated educational-clinical database to track multiple domains of case exposure and measure resident-level variation in exposure. We linked senior IM residents to patients they admitted while on overnight IM calls at 5 hospitals over 10 academic years. This study addressed 4 aims: (1) to describe the typical case exposure encountered by senior IM residents on call overnight; (2) to determine how case exposure varied between individual residents; (3) to determine how case exposure varied across 5 teaching hospitals; and (4) to determine how case exposure varied over 10 academic years.

## Methods

### Study Design, Setting, and Participants

The General Medicine Inpatient Initiative Medical Education Database (GEMINI) Medical Education Database (GEMINI-MedED) integrates education and clinical data from the University of Toronto IM residency program. GEMINI-MedED links individual senior IM residents (postgraduate year 2 or later) with patients they admitted to the IM department on overnight in-hospital call shifts. The design of GEMINI-MedED has been previously described.^20^ Briefly, GEMINI-MedED collects resident data from 5 Toronto-based teaching hospitals affiliated with the University of Toronto IM program: Mount Sinai Hospital, St Michael’s Hospital, Sunnybrook Health Sciences Centre, Toronto General Hospital, and Toronto Western Hospital. We then link resident data to patient data provided by the GEMINI database. Senior residents and patients were included based on separate inclusion and exclusion criteria (eAppendix 1 in Supplement 1). We included data starting on July 1, 2010, to match the start of data availability in GEMINI-MedEd. We selected an end date of December 30, 2019, to mitigate the impact the COVID-19 pandemic had on resident scheduling. Data analysis occurred between August 1, 2023, and June 30, 2024.

The GEMINI-MedED study was approved by the University of Toronto research ethics board, and approval was obtained from all 5 participating hospitals: Mount Sinai Hospital, St Michael’s Hospital, Sunnybrook Health Sciences Centre, Toronto General Hospital, and Toronto Western Hospital. Reporting followed the Strengthening the Reporting of Observational Studies in Epidemiology (STROBE) guidelines. Informed consent was not required from patients or residents by our research ethics board as this was a low-risk retrospective study for which data were deidentified, anonymized, and presented in aggregate.

### Residency Program and On-Call Structure

We selected IM for this study given the specialty’s clinical diversity and high patient volumes; approximately 40% of emergency department (ED) admissions are to IM.^21^ Furthermore, IM is primarily an inpatient, hospital-based specialty in Canada, allowing us to leverage existing hospital-based research databases to track exposure. At our institution, IM residents are primarily based at 1 hospital each academic year. Although residents rotate through the remaining teaching hospitals for subspecialty rotations, most IM rotations are at their base hospital. We selected senior IM residents on overnight IM call because at the University of Toronto and many North American IM programs, senior residents are responsible for triaging, examining, and reviewing all admissions to the IM department during overnight call shifts. Although junior learners are also involved in patient care, the senior IM resident bears final responsibility for overnight patient care and formally documenting their assessment. Furthermore, there is no in-hospital attending physician during overnight call; attending physicians are available by phone after hours for support and supervision. Thus, care provided during overnight call is highly attributable to the senior IM resident as it is a time of relative autonomy. Overnight IM call shifts start at 5 pm and last until 8 am the next day, but we used 6 pm as the start to avoid inappropriately attributing day team admissions to the overnight senior resident.

### GEMINI

GEMINI is an Ontario-based hospital research database that collects administrative (eg, demographic, diagnoses, and resource use/cost) and clinical data (eg, laboratory, radiology, and treatment data) from hospital information systems with 98% to 100% accuracy compared with manual abstraction.^21,22^ This database includes data from more than 30 hospitals across Ontario, including the 5 teaching hospitals involved in this study.

### Data Collection, Extraction, and Linkage

We collected call schedules from all 5 University of Toronto–affiliated hospitals and manually extracted the identities of senior residents leading the overnight shift along with the date and site of the shifts. Senior IM residents were linked to individual patients by the admission date, time, and site. Admission time was determined by the timing of admission order entering into the electronic health record (EHR). Overnight admissions to IM at all 5 hospitals were performed solely by residents and supervised by 1 senior resident. Senior residents were then linked to all their call shifts by their full names. Each resident was then deidentified and assigned a unique study number. Two independent personnel extracted 5% of call schedules in parallel to ensure interextractor agreement.

### Study Outcomes

Case exposure was measured through 6 domains: patient volumes, demographic characteristics, breadth of presentations, acuity, medical complexity, and social determinants of health. Operational definitions of each domain were based on GEMINI-MedED data availability. Patient volumes were defined by admissions per shift. Patient demographic characteristics included age at admission and sex identified in the EHR. Breadth involved the prevalence of the top 5 primary discharge diagnoses among all admissions; admission diagnoses were unavailable. Discharge diagnoses, based on the Canadian *International Classification of Diseases, 10th Revision* (*ICD-10-CA*), were mapped into clinically meaningful categories using the Clinical Classification Software Refined tool.^23,24^ The mapping process used by GEMINI has been previously described^24^ (eAppendix 2 in Supplement 1). Acuity was defined using 2 categories: calculated and clinical measures. Calculated measures included the modified laboratory-based acute physiology score, which predicts the probability of in-hospital death from admission laboratory values.^25,26^ Clinical measures included the proportion of admissions requiring intensive care unit (ICU) transfer and admissions leading to in-hospital mortality. Medical complexity also included calculated and clinical measures. Calculated measures included the Hospital Frailty Risk Score (a validated measure of frailty and increased hospital utilization that was only available for patients aged ≥65 years) and Charlson Comorbidity Index (a validated score that predicts all-cause 30-day postadmission mortality).^27,28^ The clinical measure was the proportion of patients admitted to any acute care hospital within the GEMINI network 30 days before the index admission; readmissions are a measure of complexity, as they are associated with increased mortality and length of stay.^29^ All calculated measures were preexisting in the GEMINI database. Social determinants of health included transfer from long-term care, disability status, neighborhood income (defined as the median individual annual pretax income of the neighborhood the patient resides in and the proportion of patients residing in one of Canada’s lowest income quintile neighborhoods), and neighborhood visible minority concentration (represented by the proportion of patients residing in one of Canada’s highest visible minority quintile neighborhoods). Neighborhood income and visible minority concentration data were derived from the 2016 census by Statistics Canada and linked to admissions at the dissemination area level by postal code.^30^ These metrics are used by Public Health Ontario for health services research.^31^ Housing, income, disability, and visible minority status are all social determinants of health, ie, nonmedical factors that influence health.^32^ Tracking social determinant measures is important, as residents from different specialties consistently report a lack of comfort when caring for marginalized patients.^33,34^ Increased case exposure and formal in-residency training improve practice confidence.^35^

### Statistical Analysis

For the first aim, we used descriptive statistics to describe the typical case exposure encountered by IM residents. For the second aim, we conducted resident-level analyses, dividing residents into quartiles for each case exposure measure. The standardized mean difference (SMD) between the highest and lowest exposure quartiles was calculated, with SMD values greater than 0.10 indicating meaningful imbalance between groups.^36^ For continuous measures, SMD was calculated by taking the difference in means divided by the pooled SD.^37^ For binary variables, SMD was calculated by taking the difference in proportions divided by the pooled SD. We used SMD instead of statistical hypothesis testing because it is a measure of effect size and avoids problems stemming from multiple comparisons. In an exploratory analysis to control for population-level changes in patient volumes, acuity, and complexity over time,^21,38^ residents were stratified into 3 groups by the date of their first shift in GEMINI-MedED (July 1, 2010, to June 30, 2013; July 1, 2013, to June 30, 2016; and July 1, 2016, to December 31, 2019) and the same analysis was run within each time subgroup. The date of their first shift was a surrogate for the start of their training at the senior resident level. In a second exploratory analysis, the resident cohort was restricted to those with: (1) at least 7 shifts (the number of shifts in a full academic block); (2) shifts at at least 2 hospitals; and (3) shifts across at least 2 academic years to mitigate the impact of residents with small sample sizes. Each criterion was applied separately and three separate analyses were performed.

For the third aim, we compared the hospital with the lowest proportion for each case exposure with the hospital with the highest proportion. Like aim 2, SMD was used to compare differences between hospitals. Finally, for aim 4, we used regression analysis to evaluate changes in case exposure over the 10 academic years. Linear regression was performed for continuous variables and logistic regression for categorical variables. Statistical significance was set at *P* < .05. No corrections for multiple comparisons were made and all statistical tests were presented. Analyses were performed using R version 4.3.1 (R Project for Statistical Computing).

## Results

### Resident Population

A total of 793 senior IM residents were included in GEMINI-MedED. The median (IQR) number of shifts per resident was 23 (11-28), resulting in a total of 16 231 shifts in the database. Interextractor agreement was 97% for the 889 shifts extracted in parallel (eTable 1 in Supplement 1). eFigure 1 in Supplement 1 illustrates the shifts per resident, shifts per year per resident, and admissions per resident in GEMINI-MedED. eFigure 2 in Supplement 1 illustrates the sites per resident and academic years per resident.

### Aim 1: Patient Population

Between July 1, 2010, and December 31, 2019, GEMINI-MedED included 143 632 admissions involving 88 633 unique patients. Table 1 summarizes all admissions in GEMINI-MedED. Residents encountered a median (IQR) of 8 (6-10) admissions per shift. The median (IQR) age of patients admitted was 71 (55-83) years, with 71 340 admissions (49.7%) involving female patients. The top 5 conditions encountered were pneumonia (7552 [5.3%]), congestive heart failure (CHF) (7062 [4.9%]), chronic obstructive pulmonary disease (COPD) (6112 [4.3%]), urinary tract infection (UTI) (5896 [4.1%]), and neurocognitive disorders (4180 [2.9%]), accounting for a combined 21.8% of all admissions. In terms of acuity, 7975 admissions (5.6%) resulted in in-hospital death, and 8804 (6.1%) resulted in an ICU transfer after admission. For medical complexity, 18 089 admitted patients (12.6%) were discharged within the previous 30 days from a hospital in the GEMINI network. A total of 45 921 (32.0%) had a Charlson Comorbidity Index of 2 or greater, indicating a 10-year survival of less than 90%. Finally, for social determinants of health, 11 053 admissions (7.7%) were from long-term care. The median (IQR) annual pretax individual neighborhood income of admissions was $45 444 ($38 971-$65 876), and 42 729 admissions (29.7%) resided in a low-income quintile neighborhood.

**Table 1.** Case Exposure of Admissions Managed by Senior IM Residents During Overnight Call Shifts in an IM Residency Program

Domain	Admissions, No. (%) (N = 143 632)
Volume	
Unique patients, No.	88 633
On-call shifts, No.	16 231
Admissions per shift, median (IQR)	8 (7-9)
Demographic characteristics	
Age, y	
Median (IQR)	71 (55-83)
<50	26375 (18.4)
50-75	57813 (40.3)
>75	59444 (41.4)
Sex	
Male	72290 (50.3)
Female	71340 (49.7)
Breadth (top 5 primary discharge diagnoses)	
Pneumonia	7552 (5.3)
Heart failure	7062 (4.9)
Chronic obstructive pulmonary disease	6112 (4.3)
Urinary tract infections	5896 (4.1)
Neurocognitive disorders	4180 (2.9)
Acuity	
Modified laboratory-based acute physiology score, median (IQR)^a^	15 (6-26)
In-hospital death	
Total	7975 (5.6)
Within 48 h	1208 (0.8)
Within 7 d	3477 (2.4)
Critical care transfer	
Total	8804 (6.1)
Within 48 h	5663 (3.9)
Within 7 d	7341 (5.1)
Medical complexity	
Previous 30-d hospitalization	18 089 (12.6)
Hospital Frailty Risk score^b^	
Low (≤4)	84 513 (58.8)
Intermediate (5–14)	52 282 (36.4)
High (≥15)	5516 (3.8)
Missing	1321 (0.9)
Charlson Comorbidity Index score^b^	
0	75 041 (52.2)
1	22 670 (15.8)
≥2	45 921 (32.0)
Social determinants	
Long-term care resident	11 053 (7.7)
Disability per *ICD-10* code	26 247 (18.3)
Resides in highest quintile visible minority neighborhood	28 207 (19.6)
Residents in lowest income quintile neighborhood	42 729 (29.7)
Annual neighborhood pretax individual income, median (IQR), $	51 420 (38 971-65 876)

Abbreviations: *ICD-10*, *International Classification of Diseases, 10th Edition*; IM, internal medicine.

^a^ Higher score indicates greater acuity.

^b^ Higher score indicates greater medical complexity.

### Aim 2: Variation in Case Exposure Between Residents

Table 2 outlines the variation in case exposure between the lowest and highest quartiles of residents. There was substantial variation in case exposure between residents across all 6 domains. For example, examining acuity, only 684 of 25 578 admissions (2.7%) by residents in the lowest quartile resulted in ICU transfer, compared with 3071 of 30 228 admissions (10.2%) for residents in the highest quartile (SMD, 0.31). Table 3 outlines results when residents were stratified into 3 subgroups by the date of their first shift in GEMINI-MedED. Substantial variation in case exposure persisted even between residents who became senior residents within 2 years of one another. The only case exposure measure that did not have meaningful imbalance was the proportion of admissions from lowest income–quintile neighborhoods. eTable 2 in Supplement 1 outlines results from the second sensitivity analysis. Substantial variation in exposure across all 6 domains persisted even when the resident cohort was restricted to residents with at least 7 shifts (682 residents), shifts at at least 2 hospitals (649 residents), and shifts across at least 2 academic years (532 residents).

**Table 2.** Variation in Exposure to Patient Volume, Demographic Characteristics, Breadth, Acuity, Complexity, and Social Determinants Between Residents in an Internal Medicine Residency Program

Case exposure measure	Admissions handled by 793 residents, No./total No. (%)^a^		SMD
	Lowest quartile	Highest quartile	
Volume			
Admissions per shift, median (IQR)	7 (5-9)	10 (8-12)	1.0
Demographic characteristics			
Age, median (IQR), y	66 (52-80)	76 (60-85)	0.33
Sex			
Male or other	14 028/31 214 (44.9)	15 417/27 227 (56.6)	0.24
Female	12 203/28 079 (43.5)	17 019/30 896 (55.1)	0.23
Breadth (top 5 primary discharge diagnoses)			
Pneumonia	801/27 364 (2.9)	2253/29 464 (7.6)	0.21
Heart failure	595/26 011 (2.3)	2313/31 354 (7.4)	0.24
Chronic obstructive pulmonary disease	524/25 609 (2.0)	1997/30 100 (6.6)	0.23
Urinary tract infections	532/27 425 (1.9)	1775/27 220 (6.5)	0.23
Neurocognitive disorders	241/24 442 (1.0)	1502/30 311 (5.0)	0.24
Acuity			
Laboratory-based acute physiology score, median (IQR)^b^	11 (5-20)	19 (8-31)	0.57
In-hospital death			
Total	730/25 123 (2.9)	2487/30 367 (8.2)	0.23
Within 48 h	0/28109	593/33 865 (1.8)	0.19
Within 7 d	157/22 461 (0.7)	1273/31 212 (4.1)	0.22
Critical care transfer			
Total	684/25 578 (2.7)	3071/30 228 (10.2)	0.31
Within 48 h	226/23 415 (1.0)	2315/30 094 (7.7)	0.34
Within 7 d	448/24 471 (1.8)	2680/29 667 (9.0)	0.32
Medical complexity			
Previous 30-d hospitalization	2633/28 355 (9.3)	4846/28 157 (17.2)	0.24
Hospital frailty risk score ≥5^c^	5646/17 309 (32.6)	10 018/17 544 (57.1)	0.50
Proportion of CCI ≥2^c^	6813/28 165 (24.2)	13 815/33 324 (41.5)	0.37
Social determinants			
Long-term care resident	1268/29 626 (4.3)	3089/26 689 (11.6)	0.27
Disability per *ICD-10* Code	3057/25 531 (12.0)	7832/30 996 (25.3)	0.35
Residents in highest quintile visible minority neighborhood	3396/25 130 (13.5)	7553/29 613 (25.5)	0.29
Residents in lowest quintile income neighborhood	6875/28 586 (24.1)	9697/26 465 (36.6)	0.21
Annual neighborhood pretax individual income, median (IQR), $	48 268 (35 535–63 301)	54 111 (41 084–69 621)	0.01

Abbreviations: CCI, Charlson Comorbidity Index; ICD-10, *International Classification of Diseases, 10th Edition*; SMD, standardized mean difference.

^a^ Residents were grouped into quartiles based on each case exposure measure. The SMD between the lowest and highest quartiles is reported, with values greater than 0.10 indicating a meaningful level of imbalance.

^b^ Higher score indicates greater acuity.

^c^ Higher score indicates greater medical complexity.

**Table 3.** Variation in Exposure to Patient Volume, Demographic Characteristics, Breadth, Acuity, Complexity, and Social Determinants at the Resident Level When Stratified by Time^a^

Case exposure measure	Admissions from July 1, 2010, to June 30, 2013 (288 residents and 41 854 admissions)			Admissions from July 1, 2013, to June 30, 2016 (227 residents and 46 108 admissions)			Admissions from July 1, 2016, to December 31, 2019 (278 residents and 55 670 admissions)		
	Lowest quartile, No./total No. (%)	Highest quartile, No./total No. (%)	SMD	Lowest quartile, No./total No. (%)	Highest quartile, No./total No. (%)	SMD	Lowest quartile, No./total No. (%)	Highest quartile, No./total No. (%)	SMD
Volume									
Admissions per shift, median (IQR)	6 (4-8)	9 (7-11)	1.05	7 (5-9)	10 (8-12)	0.85	8 (6-10)	10 (8-12)	0.70
Demographic characteristics									
Age, median (IQR), y	66 (51-80)	77 (61-85)	0.39	67 (53-80)	75 (59-85)	0.30	66 (51-79)	75 (59-85)	0.34
Sex									
Male and other	4045/9029 (44.8)	4521/7926 (57.0)	0.25	4111/9306 (44.2)	5845/10 607 (55.1)	0.22	5559/12 154 (45.7)	5915/10 381 (57.0)	0.23
Female	3405/7926 (43.0)	4984/9029 (55.2)	0.25	4762/10 607 (44.9)	5195/9306 (55.8)	0.22	4466/10 381 (43.0)	6595/12154 (54.2)	0.23
Breadth (top 5 primary discharge diagnoses)									
Pneumonia	159/6412 (2.5)	727/9146 (7.9)	0.25	276/9224 (3.0)	753/10 761 (7.0)	0.18	356/11 273 (3.2)	818/10 438 (7.8)	0.21
Heart failure	145/6942 (2.1)	654/8593 (7.6)	0.26	243/9658 (2.5)	785/11 082 (7.0)	0.21	213/9522 (2.2)	912/12 344 (7.4)	0.24
COPD	116/6636 (1.7)	644/9138 (7.0)	0.26	227/9443 (2.4)	647/10 187 (6.4)	0.19	195/9944 (2.0)	728/11 241 (6.5)	0.23
Urinary tract infections	118/6605 (1.8)	593/8196 (7.2)	0.26	211/9846 (2.1)	631/9821 (6.4)	0.21	214/11 275 (1.9)	599/10 539 (5.7)	0.20
Neurocognitive disorders	19/4547 (0.4)	397/9184 (4.3)	0.26	105/9321 (1.1)	492/9843 (5.0)	0.23	132/9672 (1.3)	568/10 740 (5.3)	0.22
Acuity									
Laboratory-based acute physiology score, median (IQR)^b^	11 (5-21)	20 (11-33)	0.58	12 (5-21)	20 (7-32)	0.48	11 (5-20)	18 (6-30)	0.53
In-hospital death									
Total	276/7794 (3.5)	768/8394 (9.1)	0.23	268/8480 (3.2)	850/10 929 (7.8)	0.20	218/8930 (2.4)	926/12 469 (7.4)	0.23
Within 48 h	0/6662	202/9790 (2.1)	0.21	0/8227	197/11 886 (1.7)	0.18	0/13 220	189/12 472 (1.5)	0.18
Within 7 d	47/5578 (0.8)	428/8987 (4.8)	0.24	65/8076 (0.8)	430/10 967 (3.9)	0.21	53/8680 (0.6)	399/11 472 (3.5)	0.20
Critical care transfer									
Total	123/6450 (1.9)	753/10 067 (7.5)	0.27	336/9748 (3.4)	1084/10 127 (10.7)	0.29	331/10 350 (3.2)	1116/9853 (11)	0.32
Within 48 h	15/4828 (0.3)	531/9935 (5.3)	0.31	163/9744 (1.7)	793/9228 (8.6)	0.32	142/10 692 (1.3)	821/9587 (8.6)	0.34
Within 7 d	46/5049 (0.9)	650/9989 (6.5)	0.30	261/9770 (2.7)	908/9359 (9.7)	0.29	251/10 764 (2.3)	1035/10 349 (10.0)	0.32
Medical complexity									
Previous 30-d hospitalization	660/7993 (8.3)	1343/8429 (15.9)	0.24	901/9481 (9.5)	1632/10 201 (16.0)	0.20	1197/11 518 (10.4)	1793/9592 (18.7)	0.24
Hospital Frailty Risk score ≥5^c^	1683/5369 (31.3)	3158/5558 (56.8)	0.53	1847/5556 (33.2)	3657/6438 (56.8)	0.49	1670/5209 (32.1)	4111/7276 (56.5)	0.50
Proportion of CCI ≥2^c^	1753/7581 (23.1)	3668/9260 (39.6)	0.36	2305/9495 (24.3)	4523/11 172 (40.5)	0.35	2482/10 000 (24.8)	5513/12658 (43.6)	0.40
Social determinants									
Long-term care resident	453/9371 (4.8)	892/6832 (13.1)	0.29	430/10395 (4.1)	1053/9832 (10.7)	0.25	421/10230 (4.1)	1196/10996 (10.9)	0.26
Disability per *ICD-10* code	928/7631 (12.2)	2449/9394 (26.1)	0.36	1146/9266 (12.4)	2525/9758 (25.9)	0.35	972/8539 (11.4)	2789/11 740 (23.8)	0.33
Residents in highest quintile visible minority neighborhood	853/6836 (12.5)	2173/8887 (24.5)	0.31	1193/8609 (13.9)	2521/9920 (25.4)	0.27	1488/10 331 (14.4)	2611/9931 (26.3)	0.27
Resides in lowest income quintile neighborhood	1724/7419 (23.2)	2936/7945 (37.0)	0.23	2557/10 278 (24.9)	2976/8210 (36.2)	0.19	2746/11 449 (24.0)	3621/9828 (36.8)	0.22
Annual neighborhood pretax individual income, median (IQR), $	47 989 (35 888-62 630)	53 967 (40 994-69 279)	0.02	48 943 (36 044-63 414)	53 928 (40 805-69 621)	0.01	48 343 (34 984-63 414)	54 385 (41 446-69 491)	0.02

Abbreviations: CCI, Charlson Comorbidity Index; COPD, chronic obstructive pulmonary disease; *ICD-10*, *International Classification of Diseases, 10th edition*; SMD, standardized mean difference.

^a^ The resident population was divided into 3 periods based on the year of their first shift in the database. The SMD between the lowest and highest quartiles is reported, with values greater than 0.10 indicating a meaningful level of imbalance.

^b^ Higher score indicates greater acuity.

^c^ Higher score indicates greater medical complexity.

### Aim 3: Variation in Case Exposure Between Sites

Substantial variation in case exposure was seen across the 5 hospitals in all 6 case exposure domains (Table 4). Volumes varied between hospitals as residents working in hospital B had the highest admissions per shift (median [IQR], 10 [8-12]), while residents in hospital A and C had the lowest (median [IQR], 7 [5-9]) (SMD, 0.96). Residents in hospital A had the most acute admissions based on a median (IQR) modified laboratory-based acute physiology score (21 [7-34]) and 2077 admissions (8.4%) requiring subsequent ICU transfer compared with hospital E, which had the lowest median (IQR) score (11 [5-20]) and lowest ICU transfer proportion (3.5% [952 admissions]). No single hospital stood out as caring for the most complex patients, although variation still existed for each complexity measure between hospitals. Residents in hospital C cared for patients with the lowest median (IQR) neighborhood income ($47 989 [$32 342-$63 491]) and patients living in the lowest income quintile neighborhood (9539 [38.7%]). In comparison, patients admitted to hospital B had the highest median (IQR) neighborhood income ($53 200 [$40 622-$72 892]).

**Table 4.** Variations in Volume, Demographic Characteristics, Breadth, Acuity, Complexity, and Social Determinants Between Academic Hospitals in an Internal Medicine Residency Program^a^

Case exposure measure	Admissions by hospital, No. (%)					Maximum SMD
	A (n = 24 660)	B (n = 34 401)	C (n = 24 658)	D (n = 32 723)	E (n = 27 190)	
Volume						
Admissions per shift, median (IQR)	7 (5-9)	10 (8-12)	7 (5-9)	9 (7-12)	8 (6-10)	0.96
Demographic characteristics						
Age, median (IQR), y	69 (53-83)	77 (61-86)	66 (52-80)	66 (53-79)	75 (59-84)	0.42
Sex						
Male or other	11495 (46.6)	16318 (47.4)	13800 (56)	17025 (52)	13654 (50.2)	0.19
Female	13165 (53.4)	18083 (52.6)	10858 (44)	15698 (48)	13536 (49.8)	
Breadth (top 5 primary discharge diagnoses)						
Pneumonia	1269 (5.1)	1674 (4.9)	1192 (4.8)	1992 (6.1)	1425 (5.2)	0.06
Heart failure	721 (2.9)	2152 (6.3)	963 (3.9)	1442 (4.4)	1784 (6.6)	0.17
Chronic obstructive pulmonary disease	964 (3.9)	1296 (3.8)	1272 (5.2)	989 (3.0)	1591 (5.9)	0.14
Urinary tract infections	973 (3.9)	1549 (4.5)	1156 (4.7)	1134 (3.5)	1084 (4.0)	0.06
Neurocognitive disorders	768 (3.1)	1188 (3.5)	913 (3.7)	441 (1.3)	870 (3.2)	0.15
Acuity						
Laboratory-based acute physiology score, median (IQR)^b^	21 (7-34)	17 (6-27)	15 (6-26)	12 (5-21)	11 (5-20)	0.70
In-hospital death						
Total	1487 (6.0)	1590 (4.6)	1164 (4.7)	2176 (6.7)	1558 (5.7)	0.09
Within 48 h	270 (1.1)	291 (0.8)	135 (0.5)	253 (0.8)	259 (1.0)	0.06
Within 7 d	719 (2.9)	764 (2.2)	414 (1.7)	849 (2.6)	731 (2.7)	0.08
Critical care transfer						
Total	2077 (8.4)	2307 (6.7)	2088 (8.5)	1380 (4.2)	952 (3.5)	0.21
Within 48 h	1685 (6.8)	1425 (4.1)	1414 (5.7)	662 (2.0)	477 (1.8)	0.25
Within 7 d	1893 (7.7)	1910 (5.6)	1779 (7.2)	1035 (3.2)	724 (2.7)	0.22
Medical complexity						
Previous 30-d hospitalization	3051 (12.4)	3545 (10.3)	3416 (13.9)	4648 (14.2)	3429 (12.6)	0.12
Hospital Frailty Risk score ≥5^c^	10751 (43.6)	14350 (41.7)	8663 (35.1)	11671 (35.7)	13679 (50.3)	0.31
Proportion of Charlson Comorbidity Index ≥2^c^	8434 (34.2)	8744 (25.4)	5828 (23.6)	15390 (47)	7525 (27.7)	0.51
Social determinants						
Long-term care resident	2590 (10.5)	2769 (8)	1262 (5.1)	1488 (4.5)	2944 (10.8)	0.24
Disability per *ICD-10* code	3623 (14.7)	2614 (7.6)	2933 (11.9)	6029 (18.4)	8048 (29.6)	0.45
From highest quintile visible minority neighborhoods	3918 (15.9)	8865 (25.8)	5600 (22.7)	7170 (21.9)	3240 (11.9)	0.36
From lowest income quintile neighborhood	6767 (27.4)	9325 (27.1)	9539 (38.7)	9203 (28.1)	8015 (29.5)	0.25
Annual neighborhood pre-tax individual income, median (IQR), $	52 211 (40 441-66 933)	53 200 (40 662-72 892)	47 989 (32 342-63 491)	52 338 (39 778-66 933)	50 424 (21 287-60 606)	0.40

Abbreviations: *ICD-10*, *International Classification of Diseases, 10th edition*; SMD, standardized mean difference.

^a^ In this analysis, patients were not grouped by senior residents, and each admission represents a single independent unit in this analysis. The SMD between the hospitals with the highest and lowest proportions for each measure is reported, with values greater than 0.10 indicating a meaningful level of imbalance.

^b^ Higher score indicates greater acuity.

^c^ Higher score indicates greater medical complexity.

### Aim 4: Variation in Case Exposure Over Time

The Figure illustrates variation in 6 case exposure measures over 10 academic years in GEMINI-MedED; eFigure 3 in Supplement 1 illustrates the remainder of the case exposure measures. Median (IQR) admissions per shift increased from 7.6 (6.6-8.4) in 2010 to 2011 to 9.0 (7.6-10.0) in 2019 to 2020 (*P* = .04), despite the median (IQR) shifts per year for each resident not changing over time (11 [8-14]). Patient age decreased over time, from a median (IQR) of 72 (56-83) years in 2010 to 2011 to 69 (55-82) years in 2019 to 2020 (*P* < .001), while the proportion of female patients remained at 50% over 10 years (*P* = .06). The proportion of COPD admissions increased and CHF admissions decreased, while pneumonia, UTI, and neurocognitive disorder admissions remained stable. For acuity, median (IQR) modified laboratory-based acute physiology score decreased over time from 15 (5-26) in 2010 to 2011 to 12 (5-22) in 2019 to 2020 (*P* < .001), along with the proportion of in-hospital mortality from 6.5% (893 of 13 763 patients) in 2010 to 2011 to 4.6% (373 of 8188 patients) in 2019 to 2020 (*P* = .005). For complexity, both the proportion of admissions with a Charlson Comorbidity Index of 2 or greater (28.0% [2851 patients] in 2010 to 2011 vs 35.0% [2862 patients] in 2019 to 2020; *P* = .03) and prior 30-day hospitalization (10.9% [1494 patients] in 2010 to 2011 vs 15.6% [1278 patients] in 2019 to 2020; *P* < .001) significantly increased over time. Finally, for social determinants of health, the proportion of patients transferred from long-term care decreased from 9.2% (1263 patients) in 2010 to 2011 to 6.9% (562 patients) in 2019 to 2020 (*P* = .03), whereas the other remaining social determinants did not vary significantly with time.

**Figure.**
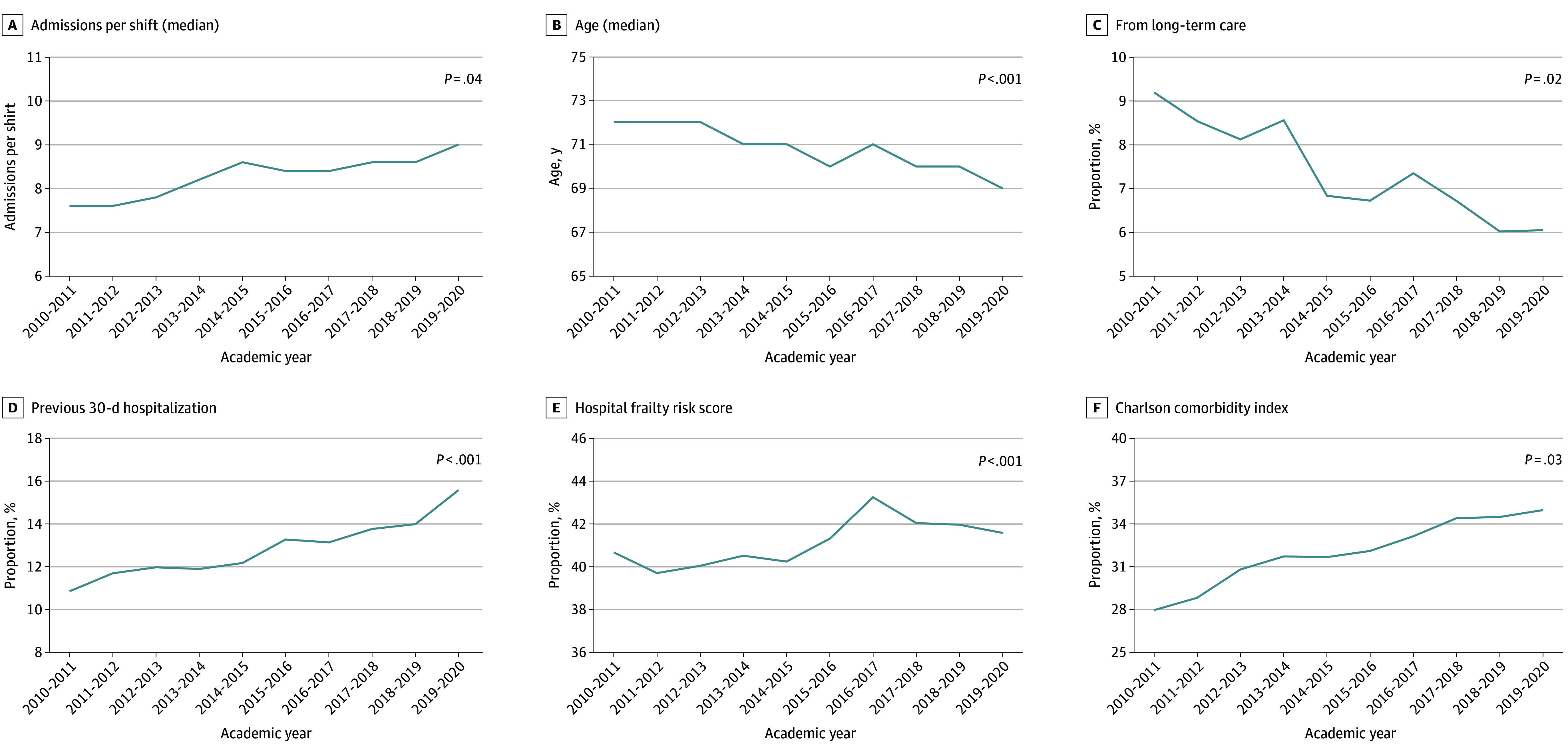
Case Exposure Measures That Significantly Varied Over the 10 Academic Years Captured in GEMINI-MedED Measures illustrated are patient volumes (A), age (B), long-term care transfers (C), previous 30-day hospitalization into a hospital within the GEMINI network (D), Hospital Frailty Risk score of 5 or greater (E), and Charlson Comorbidity Index of 2 or greater (F). Each academic year runs from July 1 until June 30 of the subsequent year. *P* values were calculated using logistic regression for categorical variables and linear regression for continuous variables to determine whether there was a significant change in that case exposure measure over time. Note, the 2019 to 2020 academic year was truncated to December 31, 2019, due to the impact of the COVID-19 pandemic on program scheduling.

## Discussion

This study characterized the case exposure of 793 senior IM residents on overnight IM calls at 5 teaching hospitals over 10 academic years in a large, urban Canadian residency program. The study leveraged an integrated education-clinical database that combined patient and resident data, resulting in 3 major findings.^20^ First, there was substantial variation in case exposure between residents in the same residency program across 6 exposure domains: volumes, demographic characteristics, breadth (as measured by discharge diagnoses), acuity, medical complexity, and social determinants. This variation was present even when examining residents who started training around the same time, suggesting population-level changes over time were not the only driver of variation. Second, there was substantial variation in case exposure across the 5 teaching hospitals, resulting in different case exposures for residents training at different sites. Finally, over the ten academic years studied (2010-2019), residents managed significantly higher volume and more complex patients, despite working a similar number of shifts per year.

We studied 2 potential sources of resident-level variation: variation over time and variation between hospitals. In our study, trainees who started residency later (2019-2020) encountered substantially more complex patients than those who started earlier (2010-2011). This likely reflects broader population-level health trends over time driven by an aging population, advancements in prescription drugs, and increased social challenges stemming from homelessness, substance use, and psychiatric illness.^39^ Notably, a study from Naik et al^38^ of approximately 3.4 million Canadian inpatient admissions between 2002 and 2017 also demonstrated a rise in medical complexity driven by increased comorbidities, polypharmacy, and readmission rates. Our study found that these population trends are also reflected in resident experiences.

The second source of variation studied was hospital-level differences, likely stemming from differing admission and referral guidelines, resources, and local patient populations.^40^ Our study had similar findings as Rhee et al,^41^ who examined discharge diagnoses from 4 hospitals, all affiliated with a single US residency program. This suggests that differences in resident training experiences between hospital sites may be a generalizable phenomenon.

This study’s findings align with the increased focus on outcomes-based approaches in specialty training, driven by the adoption of competency-based medical education.^42^ National accrediting bodies mandate that residents obtain broad exposure to the full range of their specialty to be prepared for independent practice.^43^ We demonstrated how EHR data can be harnessed to measure resident-level case exposure, ensuring that a breadth of exposure is achieved. These data could be used at multiple levels of medical education: for residents, to identify potential exposure gaps and personalize their selection of future training experiences; for hospital and residency training programs, to understand variation between sites and ensure residents have balanced exposure to diverse patients; and for residency accreditation bodies, to guide decision-making and standards regarding training requirements at a national level.

Future work planned for GEMINI-MedED includes moving beyond case exposure into tracking resident clinical decision-making and its impact on patient outcomes. Concurrently, we aim to develop a prospective system that enables real-time feedback that can be integrated into other tools of resident assessment. This is akin to attending physician audit-and-feedback systems already implemented but would take place during residency, a time of significant imprinting.^44^ Ultimately, the goal is to develop educational outcomes that are directly associated with patient outcomes and are also timely, minimize the burden on resident and educators, and offer a more comprehensive view of the residency experience.

### Limitations and Strengths

There are several limitations to this study. First, GEMINI-MedED only captured overnight admissions to IM. IM residency includes additional clinical experiences (eg, subspecialty rotations, daytime ward rounding, outpatient clinics, community hospitals) that were not captured in this study. Although resident-level variation was observed overnight, exposure through these other experiences may reduce the extent of observed variation. Second, this study was carried out in a single, urban, Canadian residency program. Differences in the location, structure, and other contextual variables may limit the generalizability of our findings to other residency programs. Regardless, this study may serve as a proof of concept of linking residents to patients through clinical datasets. Third, resident data were limited by call schedule availability, as 3 academic years were missing from hospital D, accounting for approximately 6% of shifts during the study time frame. However, missing call schedule data did not have a meaningful impact on the major findings, as resident-level variation remained unchanged in the sensitivity analyses in which our resident cohort was restricted to those for whom we had more complete data. Furthermore, our study was limited by the data availability of GEMINI. Although GEMINI contains multidimensional measures of case exposure, admission diagnoses are not available in Canadian administrative data, and the primary discharge diagnosis was used instead. Although not perfect, there is a high degree of correlation between the admission and discharge diagnoses administrative data.^45,46^

The major strength of this study was the use of GEMINI-MedED, which is based on clinical data derived from EHRs and has been previously validated for data accuracy.^22^ Unlike trainee-reported case logs or preceptor-driven evaluations, patient and resident data were derived from empirical sources with a lower risk of reporting bias and error.^47^ Furthermore, this integrated education-clinical dataset enabled us to build on previous efforts to characterize resident exposure in several ways.^7,9,13,14,41,48^ First, clinical data collected in this study were multidimensional and also included measures of social determinants of health, compared with previous studies that focused on a single dimension of exposure.^3^ Although marginalized populations experience worse health outcomes, trainee exposure to diverse patient populations is associated with improved perceived preparedness to care for them.^49,50^ Therefore, case exposure data on the social determinants of patients may also inform educational design to ensure trainees are exposed to both diverse medical conditions and patient populations. Second, this study derived data from multiple sites and academic years, providing a more comprehensive view of the IM residency experience. Additionally, and most importantly, we attributed residents to individual patients using call schedule data, enabling us to perform resident-level analyses, moving beyond previous aggregate-level studies.^3^

## Conclusions

In this cohort study of IM residents, we found substantial variation in each resident’s case exposure even within a single residency program. Sources of variation included differences in patient populations between hospitals and academic years, among other factors. This study demonstrated residency case exposure can be tracked through an integrated educational-clinical database. Understanding how case exposure varies within programs may have relevance at multiple levels of medical education, including residents, hospitals, residency programs, and accrediting organizations.

## Notes

Supplement 1. **eFigure 1. **Characteristics of Overnight Internal Medicine Calls in the General Medicine Inpatient Initiative Medical Education Database (GEMINI MedED) **eFigure 2. **Characteristics of Residents’ Overnight Internal Medicine Call in the General Medicine Inpatient Initiative Medical Education Database (GEMINI MedED) **eFigure 3. **Line Graphs of Patient Volumes, Demographic Characteristics, Breadth, Acuity, Medical Complexity, and Social Determinants Over 10 Academic Years **eTable 1.** General Medicine Inpatient Initiative Medical Education Database Resident Inventory **eTable 2.** Variation in Exposure to Patient Volume, Demographic Characteristics, Breadth, Acuity, Complexity, and Social Determinants Between Residents in an Internal Medicine Residency Program **eAppendix 1. **GEMINI-MedED Inclusion and Exclusion Criteria **eAppendix 2. **GEMINI Clinical Classification Software Refined (CCSR) Code

Supplement 2.
Data Sharing Statement
