# Supplementary material for: Variation in Case Exposure During Internal Medicine Residency
Source: JAMA Netw Open. 2024 Dec 18;7(12):e2450768. doi: 10.1001/jamanetworkopen.2024.50768 (PMC11656263; doi:10.1001/jamanetworkopen.2024.50768)
Supplement: Supplement 1. — eFigure 1. Characteristics of Overnight Internal Medicine Calls in the General Medicine Inpatient Initiative Medical Education Database (GEMINI MedED) eFigure 2. Characteristics of Residents’ Overnight Internal Medicine Call in the General Medicine Inpatient Initiative Medical Education Database (GEMINI MedED) eFigure 3. Line Graphs of Patient Volumes, Demographic Characteristics, Breadth, Acuity, Medical Complexity, and Social Determinants Over 10 Academic Years eTable 1. General Medicine Inpatient Initiative Medical Education Database Resident Inventory eTable 2. Variation in Exposure to Patient Volume, Demographic Characteristics, Breadth, Acuity, Complexity, and Social Determinants Between Residents in an Internal Medicine Residency Program eAppendix 1. GEMINI-MedED Inclusion and Exclusion Criteria eAppendix 2. GEMINI Clinical Classification Software Refined (CCSR) Code [file jamanetwopen-e2450768-s001.pdf]

Supplemental Online Content

Lam AL, Tang B, Liu C, et al. Variation in case exposure during internal medicine residency. *JAMA Netw Open.* 2024;7(12):e2450768. doi:10.1001/jamanetworkopen.2024.50768

eFigure 1. Characteristics of Overnight Internal Medicine Calls in the General Medicine Inpatient Initiative Medical Education Database (GEMINI MedED)

eFigure 2. Characteristics of Residents' Overnight Internal Medicine Call in the General Medicine Inpatient Initiative Medical Education Database (GEMINI MedED)

eFigure 3. Line Graphs of Patient Volumes, Demographic Characteristics, Breadth, Acuity, Medical Complexity, and Social Determinants Over 10 Academic Years

eTable 1. General Medicine Inpatient Initiative Medical Education Database Resident Inventory

eTable 2. Variation in Exposure to Patient Volume, Demographic Characteristics, Breadth, Acuity, Complexity, and Social Determinants Between Residents in an Internal Medicine Residency Program

eAppendix 1. GEMINI-MedED Inclusion and Exclusion Criteria

eAppendix 2. GEMINI Clinical Classification Software Refined (CCSR) Code

This supplemental material has been provided by the authors to give readers additional information about their work.

© 2024 Lam AL et al. *JAMA Network Open.*

eFigure 1

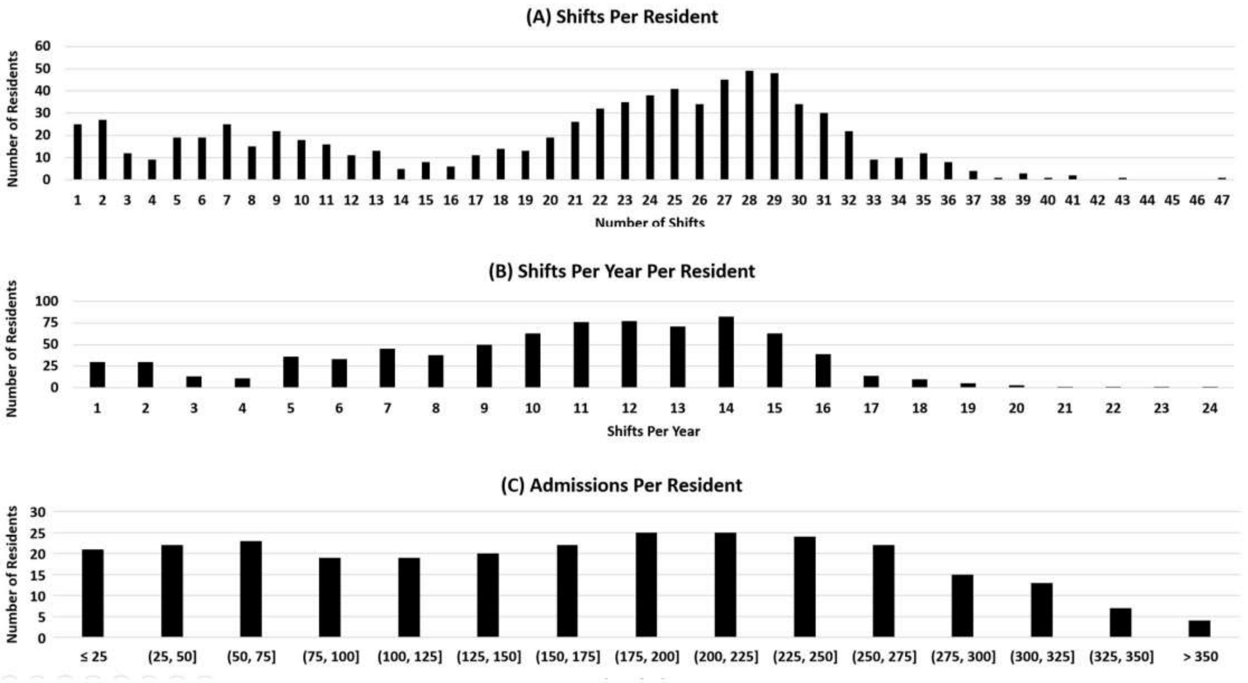

eFigure 1. Characteristics of Overnight Internal Medicine Calls in the General Medicine Inpatient Initiative Medical Education Database (GEMINI MedED)

Panel A outlines the shifts per resident, panel B outlines the shifts per year per resident, and panel C outlines the number of admissions per resident during their residency captured by GEMINI MedED.

© 2024 Lam AL et al. *JAMA Network Open.*

eFigure 2

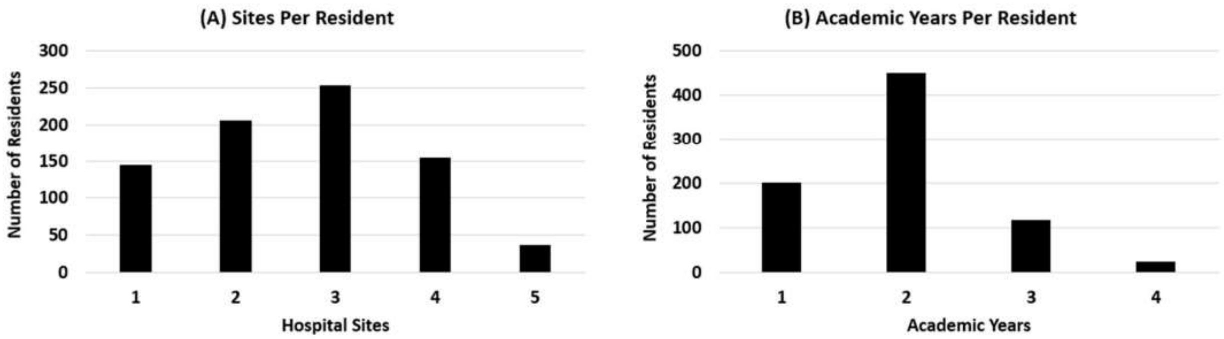

eFigure 2. Characteristics of Residents' Overnight Internal Medicine Call in the General Medicine Inpatient Initiative Medical Education Database (GEMINI MedED)

Panel A illustrates the number of distribution of sites worked per resident and panel B illustrates the distribution of academic years worked per resident.

© 2024 Lam AL et al. *JAMA Network Open.*

eFigure 3

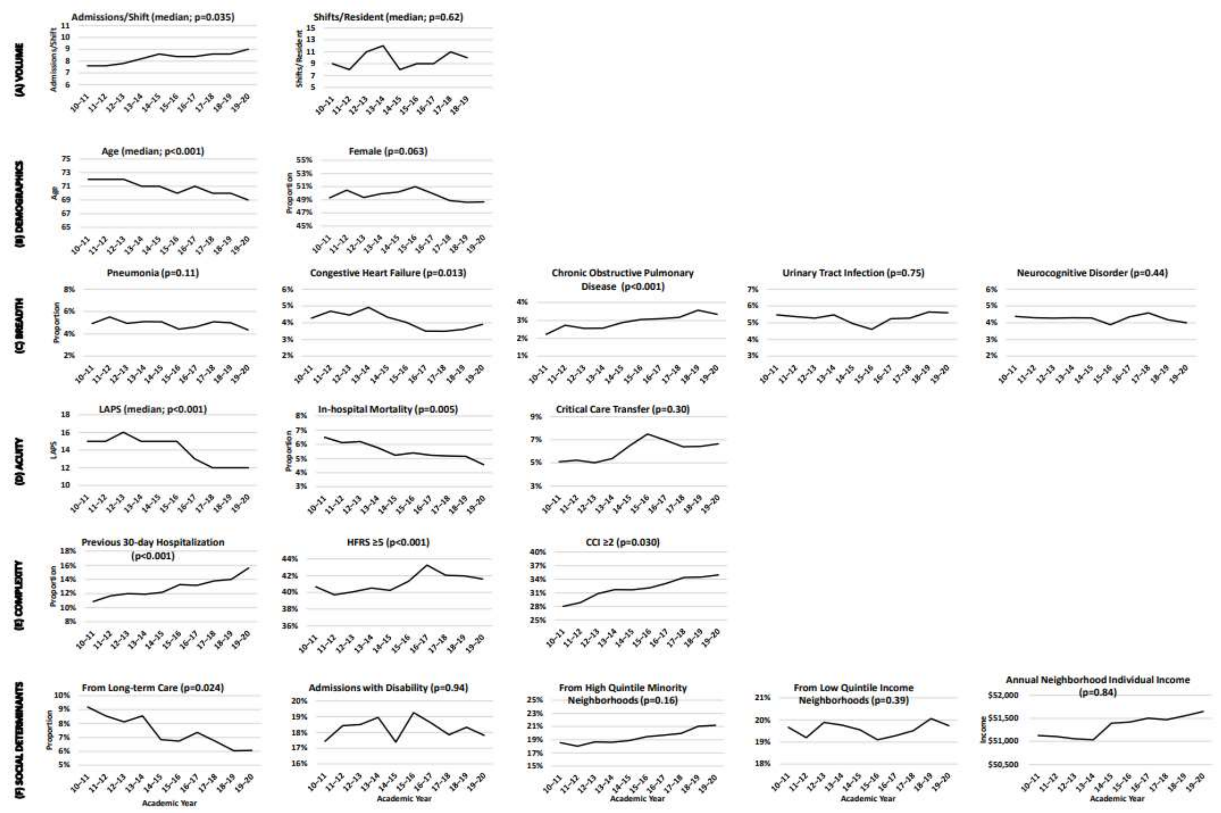

eFigure 3. Line Graphs of Patient Volumes (row A), Demographic Characteristics (row B), Breadth (row C), Acuity (row D), Medical Complexity (row E), and Social Determinants (row F) Over 10 Academic Years

Each academic year runs from July 1st until Jun 30th of the subsequent year. P-values were calculated using logistic regression for categorical variables and linear regression for continuous variables to determine if there was a significant change in that case exposure variable over time. Note that the 2019–2020 academic year was truncated to December 31st, 2019, due to the impact of COVID-19 on program scheduling. Thus, shifts per resident did not include the 2019–2020 academic year. Abbreviations: LAPS, lab-based acute physiology score; HFRS, hospital frailty risk score; CCI, Charlson Comorbidity Index.

© 2024 Lam AL et al. *JAMA Network Open.*

eTable 1. General Medicine Inpatient Initiative Medical Education Database Resident Inventory

Site	Date Range	Total Shifts	Unique Residents	Median Shifts per Resident [IQR]	Interrater Agreement (%) (n extracted in parallel) [a]
**A**	Jul 1, 2010 – Dec 31, 2019	3471	394	7 [2–15]	94% (214)
**B**	Jul 1, 2012 – Jun 30, 2014<br>Jul 1, 2015 – Dec 31, 2019	2375	320	5 [1–12]	100% (154)
**C**	Jul 1, 2010 – Dec 31, 2019	3471	484	7 [(2–11]	97% (175)
**D**	Jul 1, 2010 – Dec 31, 2019 [b]	3443	487	7 [2–11]	97% (180)
**E**	Jul 1, 2010 – Dec 31, 2019	3471	426	6 [2–15]	98% (166)
**Total**	**Jul 1, 2010 – Dec 31, 2019**	**16,231**	**793**	**23 [11–28]**	**97% (889)**

[a] Five percent independently extracted in parallel

[b] Missing November 1–28, 2010, inclusive

Abbreviations: IQR, interquartile range.

© 2024 Lam AL et al. *JAMA Network Open.*

eTable 2. Variation in Exposure to Patient Volume, Demographic Characteristics, Breadth, Acuity, Complexity, and Social Determinants Between Residents in an Internal Medicine Residency Program
Exploratory analyses were conducted within subgroups of the resident population, excluding those with a minimal number of shifts, sites, and academic years recorded in the database. The standardized mean difference (SMD) between the lowest and highest quartiles was reported, with bolded values indicating a meaningful level of imbalance.

Case Exposure Measure	**Residents with ≥7 Shifts** (n=682)			**Residents Working in ≥2 Sites** (n=649)			**Residents Across ≥2 Academic Years** (n=532)		
	**Lowest Quartile**	**Highest Quartile**	**SMD**	**Lowest Quartile**	**Highest Quartile**	**SMD**	**Lowest Quartile**	**Highest Quartile**	**SMD**
**Volume**									
Admissions per Shift (median [IQR])	7 [5–9]	10 [8–12]	**0.99**	7 [5–9]	10 [8–2]	**0.98**	7 [5–9]	10 [8–12]	**0.97**
**Demographics**									
Age (median years [IQR])	66 [52–80]	75 [59–85]	**0.30**	67 [52–80]	75 [59–85]	**0.30**	67 [52–80]	75 [59–85]	**0.30**
Male/Other Proportion (%)	45%	56%	**0.22**	45%	56%	**0.21**	45%	56%	**0.21**
Female Proportion (%)	44%	55%	**0.22**	44%	55%	**0.21**	44%	55%	**0.21**
**Breadth**									
Top 5 Discharge Diagnoses									
#1 Pneumonia	3.2%	7.5%	**0.19**	3.1%	7.4%	**0.19**	3.2%	7.3%	**0.19**
#2 Heart failure	2.5%	7.3%	**0.22**	2.6%	7.3%	**0.22**	2.7%	7.2%	**0.21**
#3 Chronic obstructive pulmonary disease	2.3%	6.5%	**0.21**	2.3%	6.5%	**0.21**	2.5%	6.4%	**0.19**
#4 Urinary tract infections	2.1%	6.2%	**0.21**	2.1%	6.3%	**0.21**	2.2%	6.2%	**0.20**
#5 Neurocognitive disorders	1.2%	4.8%	**0.22**	1.2%	4.9%	**0.22**	1.3%	4.8%	**0.21**
**Acuity**									
Lab-based Acute Physiology Score (median [IQR]) [a]	11 [5–21]	19 [8–31]	**0.49**	11 [5–21]	19 [8–31]	**0.48**	11 [5–21]	19 [8–31]	**0.47**
Total In-hospital death (%)	3.2%	8.1%	**0.21**	3.3%	8.1%	**0.21**	3.3%	8.0%	**0.20**
Within 48 hours	0%	1.7%	**0.19**	0%	1.7%	**0.19**	0%	1.7%	**0.18**
Within 7 days	0.9%	4.0%	**0.20**	0.9%	4.0%	**0.20**	0.9%	4.0%	**0.19**
Total Critical Care Transfer (%)	3.0%	10%	**0.29**	3.0%	9.9%	**0.28**	3.1%	10%	**0.28**
Within 48 hours	1.2%	7.6%	**0.31**	1.2%	7.4%	**0.31**	1.3%	7.5%	**0.30**
Within 7 days	2.2%	8.9%	**0.30**	2.1%	8.8%	**0.30**	2.2%	8.8%	**0.29**
**Medical Complexity**									
Previous 30-day Hospitalization (%)	9.5%	17%	**0.22**	9.4%	17%	**0.22**	9.6%	17%	**0.21**
Hospital Frailty Risk Score ≥5 (%) [b]	34%	57%	**0.47**	34%	57%	**0.46**	35%	57%	**0.45**
Proportion of Charlson Comorbidity Index ≥2 (%) [b]	24%	41%	**0.36**	25%	41%	**0.35**	25%	40%	**0.34**
**Social Determinants**									
Long-term Care Resident (%)	4.4%	11%	**0.26**	4.5%	11%	**0.25**	4.7%	11%	**0.24**
Disability per ICD-10 Code (%)	12%	25%	**0.32**	13%	25%	**0.32**	13%	25%	**0.31**
From Highest Quintile Visible Minority Neighborhoods (%)	14%	25%	**0.27**	14%	25%	**0.27**	14%	25%	**0.25**
From Lowest Income Quintile Neighborhood (%)	24%	36%	**0.20**	25%	36%	**0.19**	25%	36%	**0.19**
Annual Neighborhood Pre-tax Individual Income (median [IQR])	$48,347 [$27,471]	$53,928 [$28,604]	0.01	$48,473 [$27,292]	$53,928 [$28,623]	0.01	$48,569 [$27,384]	$53,928 [$28,471]	0.01

[a] Higher score indicates greater acuity
[b] Higher score indicates greater medical complexity
Abbreviations: SMD, standardized mean difference; IQR, interquartile range; ICD-10, International Classification of Diseases, 10th Revision.

© 2024 Lam AL et al. *JAMA Network Open.*

eAppendix 1. GEMINI-MedED Inclusion and Exclusion Criteria

Inclusion Criteria Resident

a) Post-graduate year 2, 3, 4, or 5 (senior residents)

b) Enrolled in the University of Toronto core IM Program

c) One or more IM overnight call shifts as a senior resident at one of the five participating teaching hospitals

d) Date of call shift between July 1, 2010, until December 30, 2019

Exclusion Criteria Resident

a) Visiting or elective resident enrolled in another post-graduate training program

b) Post-graduate year 1 (junior residents)

Inclusion Criteria Patient

a) Over the age of 18

b) Admitted to Internal Medicine through the emergency department at one of five participating hospitals (i.e., Mount Sinai Hospital, St. Michael's Hospital, Sunnybrook Health Sciences Centre, Toronto General Hospital, and Toronto Western Hospital)

c) Date of admission between July 1, 2010, and December 30, 2019

d) Time of Admission between 6 PM until 8 AM the following day

Exclusion Criteria Patient

a) Admissions/transfers not through the emergency department (e.g., surgical, subspecialty, or intensive care unit transfers, elective admissions)

b) Admission to a general medicine or hospitalist team not supervised by an internist (e.g. family medicine hospitalist service)

© 2024 Lam AL et al. *JAMA Network Open.*

eAppendix 2. GEMINI Clinical Classification Software Refined (CCSR) Code

https://github.com/GEMINI-Medicine/gemini-ccsr
