# Supplementary material for: Variation in Case Exposure During Internal Medicine Residency
Source: JAMA Netw Open. 2024 Dec 18;7(12):e2450768. doi: 10.1001/jamanetworkopen.2024.50768 (PMC11656263; doi:10.1001/jamanetworkopen.2024.50768)
Supplement: Supplement 2. — Data Sharing Statement [file jamanetwopen-e2450768-s002.pdf]

Data Sharing Statement

Lam. Variation in Case Exposure During Internal Medicine Residency. *JAMA Netw Open*. Published December 13, 2024. doi:10.1001/jamanetworkopen.2024.50768

Data
Data available: No
